# Interplay between mitochondrial dysfunction and lysosomal storage: challenges in genetic metabolic muscle diseases with a focus on infantile onset Pompe disease

**DOI:** 10.3389/fcvm.2024.1367108

**Published:** 2024-02-20

**Authors:** Mengjiao Zhang, Jiechao Niu, Mengmeng Xu, Erhu Wei, Peng Liu, Guangyao Sheng

**Affiliations:** Department of Pediatrics, The First Affiliated Hospital of Zhengzhou University, Zhengzhou, China

**Keywords:** hereditary myopathy, lysosomal storage, Pompe disease, enzyme replacement therapy, alpha-glucosidase

## Abstract

**Background:**

Pompe disease (PD) is a rare, progressive autosomal recessive lysosomal storage disorder that directly impacts mitochondrial function, leading to structural abnormalities and potentially culminating in heart failure or cardiogenic shock. The clinical course and molecular mechanisms of the disease remain incompletely understood.

**Methods:**

We performed a retrospective analysis to examine the clinical manifestations, genetic traits, and the relationship between PD and mitochondrial function in a pediatric patient. This comprehensive evaluation included the use of ultrasound echocardiograms, computed tomography (CT) scans, electrocardiograms, mutagenesis analysis, and structural analysis to gain insights into the patient's condition and the underlying mechanisms of PD. For structural analysis and visualization, the structure of protein data bank ID 5KZX of human GAA was used, and VMD software was used for visualization and analysis.

**Results:**

The study revealed that a 5-month-old male infant was admitted due to fever, with physical examination finding abnormal cardiopulmonary function and hepatomegaly. Laboratory tests and echocardiography confirmed heart failure and hypertrophic cardiomyopathy. Despite a week of treatment, which normalized body temperature and reduced pulmonary inflammation, cardiac abnormalities did not show significant improvement. Further genetic testing identified a homozygous mutation c.2662G>T (p.E888) in the GAA gene, leading to a diagnosis of Infantile-Onset Pompe Disease (IOPD).

**Conclusions:**

Although enzyme replacement therapy can significantly improve the quality of life for patients with PD, enhancing mitochondrial function may represent a new therapeutic strategy for treating PD.

## Introduction

Pompe disease (PD), also known as Glycogen Storage Disease Type II (OMIM: 232300), or Acid Alpha-Glucosidase Deficiency, is a rare inherited lysosomal storage disorder. It typically manifests in newborns up to three months after birth, progressing rapidly with varied clinical symptoms. These include hypotonia, feeding difficulties, developmental delays, and progressive myocardial hypertrophy, leading to heart and respiratory failure in untreated children (1). The disease causes abnormal glycogen accumulation and increased production of reactive oxygen species (ROS) (2). Studies suggest that interactions between lysosomes and mitochondria can significantly affect cellular function. In PD, this may indirectly impact mitochondrial function through mechanisms like autophagy, disturbed calcium balance, cellular signaling disruptions, and metabolic product exchange. This can lead to structural abnormalities in mitochondria, resulting in energy deficiency and muscle contraction failure (3, 4). Early diagnosis is crucial for improving survival rates and quality of life; however, its rarity and non-specific symptoms often make diagnosis challenging (1). Clinical diagnosis relies on measuring GAA enzyme activity and analyzing GAA gene mutations. The most effective current treatment is enzyme replacement therapy (ERT) centered around recombinant human acid alpha-glucosidase (rhGAA). This study aims to analyze the clinical characteristics, diagnostic approach, and current treatment strategies for a five-month-old male infant with PD, providing references for future research and clinical practice.

## Case presentation

A 5-month-old male infant was admitted to the Pediatrics Department of The First Affiliated Hospital of Zhengzhou University on March 14, 2022. The admission was due to a fever that lasted for four days. The peak temperature reached 40°C, with no symptoms of cough, wheezing, or breathing difficulties, and no vomiting, consciousness disorder, or convulsions. The infant was a first-born, delivered full-term, with an unremarkable perinatal period. The birth weight was 3.25 kg. He was fed a combination of breast milk and formula, but experienced feeding difficulties. At 4 months, he had unstable head control, and by 5 months, he could roll over. Upon admission, the physical examination showed: temperature 38.4°C, heart rate 168 beats per minute, respiratory rate 40 per minute, blood pressure 112/69 mmHg, height 61 cm, weight 6.4 kg, head circumference 41 cm, chest circumference 40.5 cm, abdominal circumference 38 cm. The infant exhibited growth and developmental delays, coarse breathing sounds in both lungs with audible wet rales, fast heart rate, muffled heart sounds, regular rhythm, no audible murmurs at valve auscultation areas, enlarged liver palpable 5 cm below the right costal margin and 3 cm below the xiphoid process, spleen not palpable, and reduced muscle tone.

Laboratory tests revealed: pH 7.46 (normal 7.35–7.45), carbon dioxide pressure 39.0 mmHg (35–48 mmHg), oxygen pressure 69.0 mmHg (83–108 mmHg), residual base 3.90 mmol/L (−2 to 3 mmol/L), glucose 6.0 mmol/L (3.3–5.3 mmol/L), white blood cell count 19.15 × 10^9^/L (3.5–9.5 × 10^9^/L), hemoglobin 80.0 g/L (120–150 g/L), platelet count 437 × 10^9^/L (125–350 × 10^9^/L), neutrophil percentage 45.4% (40%–75%), lymphocyte percentage 47.0% (20%–50%); procalcitonin 0.954 ng/ml (0–0.046 ng/ml), C-reactive protein 111.41 mg/L (0–5 mg/L), interleukin-6 9.56 pg/ml (0–7 pg/ml); calcium 2.40 mmol/L (2.5–3 mmol/L), phosphorus 0.89 mmol/L (1.29–2.26 mmol/L), alanine aminotransferase 189 U/L (0–40 U/L), aspartate aminotransferase 206 U/L (0–40 U/L), creatine kinase 972 U/L (39–308 U/L), creatine kinase isoenzyme 52.9 U/L (0–25 U/L), lactate dehydrogenase 1,317 U/L (200–600 U/L), hypersensitive troponin T 0.124 ng/ml (0–0.014 ng/ml); N-terminal pro b-type natriuretic peptide 10,066.50 pg/ml (0–97.3 pg/ml), troponin I 0.131 μg/L (0–0.034 μg/L); other lab results were not significantly abnormal.

**Figure 1:**
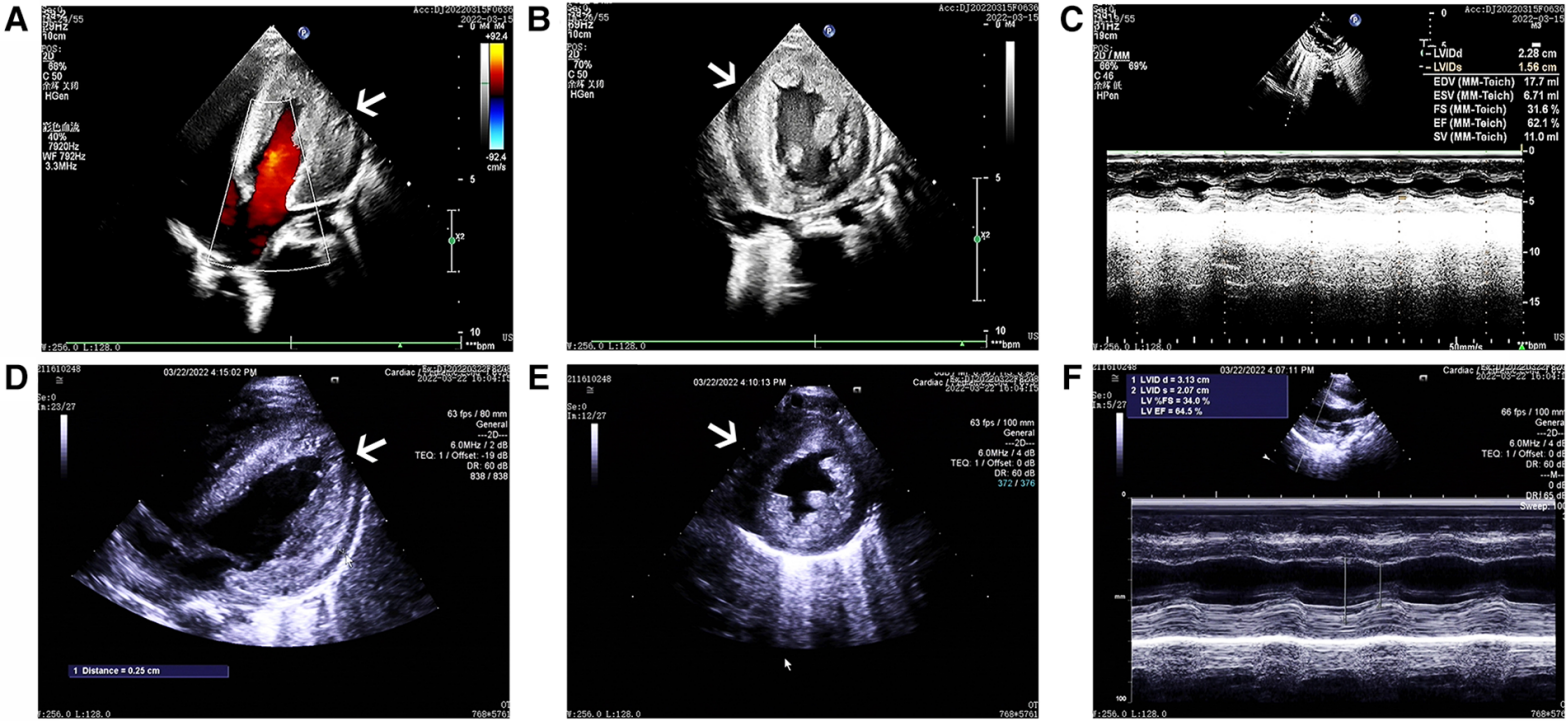
The ultrasound echocardiograms of the patient showed significant myocardial hypertrophy. The images (**A**–**C**) sequentially represent the apical four-chamber view, the left ventricular short-axis view, and the M-mode ultrasound changes before treatment. Similarly, images (**D**–**F**) represent the same views and changes after treatment.

**Figure 2:**
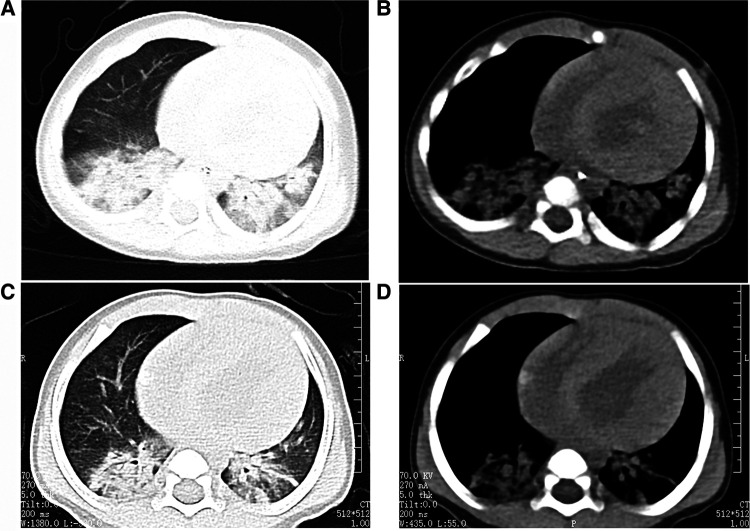
The child's chest CT scans indicated bilateral pneumonia. (**A** and **B**: before treatment, **C** and **D**: after treatment), with a significantly enlarged cardiac silhouette. After one week of treatment, the pulmonary imaging showed improvement, but there was no noticeable change in the size of the cardiac silhouette.

**Figure 3:**
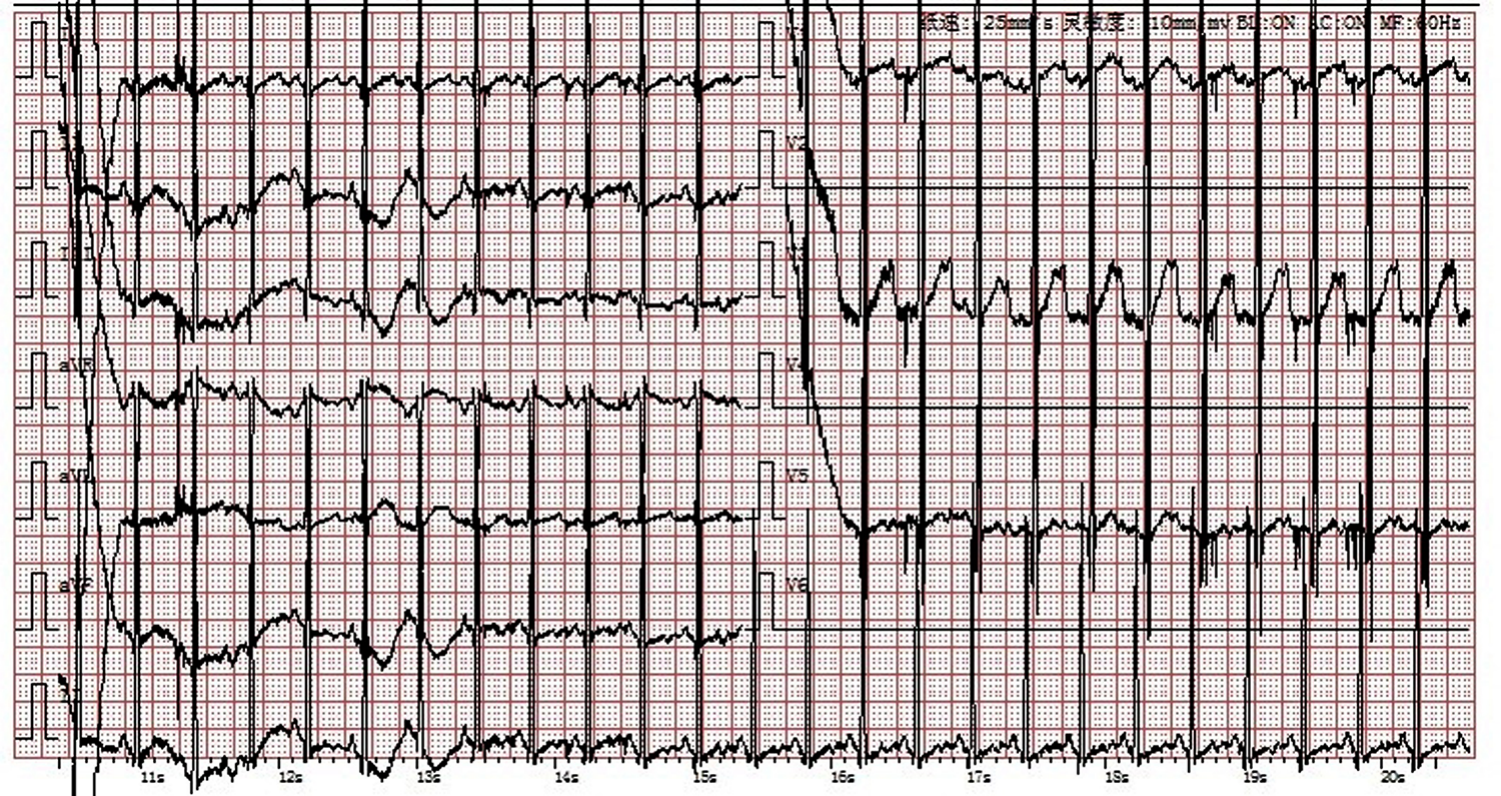
Electrocardiogram: Sinus tachycardia, enlargement of the left atrium and left ventricle with ST-T changes.

The cardiac echocardiography (Figures 1A–C) showed diffuse thickening of the left ventricular wall, with the anterior wall approximately 9.5 mm thick, the interventricular septum around 8 mm, the lateral wall about 9 mm, and the inferoposterior wall approximately 9 mm. The thickened myocardial wall had increased echogenicity and stiffness, with generally reduced movement amplitude. The endocardium of the left ventricle was thickened, up to about 5 mm. The ejection fraction (EF) was 62%, and the shortening fraction of the left ventricular short axis (FS) was 31%. Color Doppler Flow Imaging (CDFI) showed no shunt at the ventricular level. A small left-to-right shunt was observed at the middle of the interatrial septum (foramen ovale), approximately 2 mm wide. A small amount of regurgitation was noted at the mitral valve, and a trace regurgitation signal was seen at the tricuspid valve with a regurgitation speed of 1.8 m/s, estimating pulmonary artery pressure at 18 mmHg (right atrial pressure assumed to be 5 mmHg). The abdominal ultrasound showed hepatomegaly and edematous gallbladder walls. The chest CT scan revealed normal translucency in both lungs with disordered patterns, patchy areas of increased density, localized consolidation with visible air bronchograms, normal hilar shadows, and clear airways. The cardiac silhouette was enlarged, with thickening of the left ventricular myocardial wall (Figure 2A). The electrocardiogram showed sinus tachycardia, enlargement of the left atrium and left ventricle, accompanied by ST-T changes (Figure 3).

The patient was initially diagnosed with severe pneumonia combined with heart failure and hypertrophic cardiomyopathy due to fever, significant increase in white blood cells and C-reactive protein (CRP), and definitive chest imaging. Treatment was initiated with cefoperazone and acyclovir for infection control and nebulized medication to reduce airway inflammation, along with hepatoprotective drugs. Fluid management was emphasized, with the use of low-dose diuretics (hydrochlorothiazide and spironolactone) to improve heart failure and hepatomegaly caused by excessive circulatory load. Considering the fast heart rate and reduced left ventricular systolic and diastolic function, the patient was treated with propranolol to slow down the heart rate, reduce left ventricular contractility and wall stress, and improve diastolic function. Benazepril was also administered to mitigate myocardial remodeling.

After one week of regular anti-infection treatment, liver protection, and heart function improvement, the infant's body temperature normalized, and a recheck showed a significant decrease in N-terminal pro-brain natriuretic peptide (1,730.94 pg/ml). CT scans indicated reduced lung inflammation (Figure 2B). However, a follow-up cardiac echocardiography (Figures 1D–F) still indicated symmetric hypertrophic cardiomyopathy (non-obstructive type) with thickening of the left ventricular endocardium, and decreased left ventricular systolic and diastolic function (EF 48%, FS 22%). The liver size had not noticeably reduced, and there was no improvement in the liver function tests (alanine aminotransferase and aspartate aminotransferase) compared to admission. Serum creatine kinase levels were 476 U/L, which was inconsistent with clinical expectations. This lack of noticeable improvement in the echocardiographic images underscores the severity and progression of the disease, despite the treatment efforts.

**Figure 4:**
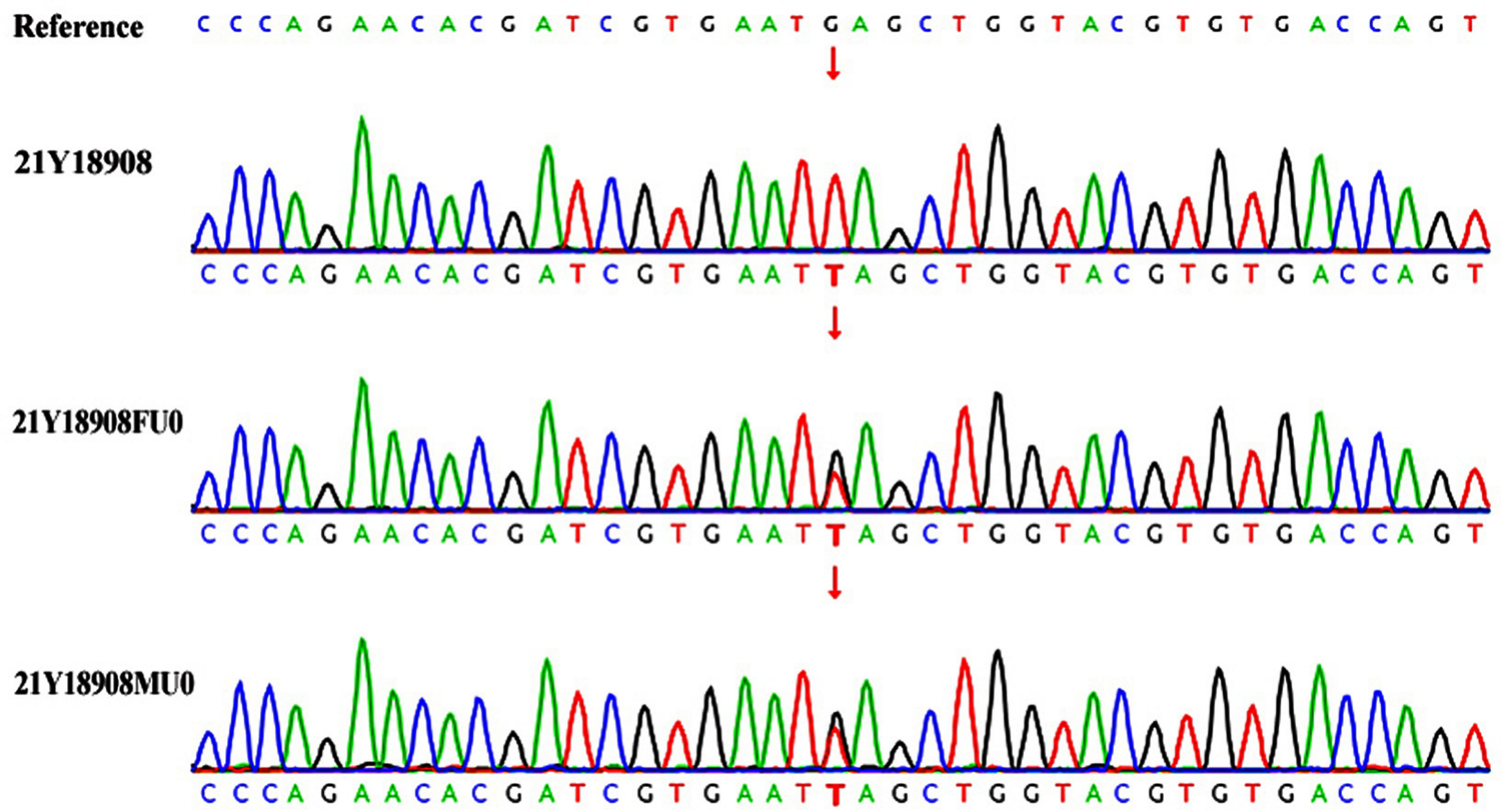
Genetic testing 21Y18908—index case; 21Y18908FU0—father; 21Y18908MU0—mother. The child's GAA gene has a homozygous mutation c.2662G>T, with both parents being carriers of the genetic mutation.

Therefore, considering the young age of onset, liver enlargement at admission, myocardial hypertrophy, abnormal serum creatine kinase spectrum, liver function abnormalities, and significant developmental delays, the possibility of a genetic metabolic disease was considered. Blood organic acid metabolic screening and family exome genetic testing were conducted for the patient. The blood organic acid metabolic screening showed no abnormalities, but genetic testing revealed a homozygous mutation c.2662G>T(p.E888) in the GAA gene in the infant, with both parents being carriers of this mutation site (Figure 4). According to the ACMG guidelines, this gene mutation is pathogenic. Further testing for GAA enzyme activity in the patient showed reduced activity. Combining the clinical presentation, physical examination, and related test results, the infant was ultimately diagnosed with Infantile-Onset Pompe Disease (IOPD). Due to the high cost of rhGAA, the enzyme replacement therapy, the family decided against it and requested discharge. Regrettably, follow-up information on the patient's condition after discharge was not successfully obtained.

## Discussion

### Concurrent discovery of IOPD and cardiac disease

In this study, we report a unique case. The infant was diagnosed with Infantile-Onset Pompe Disease (IOPD) during a visit for an infectious disease. Additionally, myocardial disease was detected in the infant. This is uncommon in cases of IOPD. Research and statistical analysis show that the median age of symptom onset for IOPD is 2.0 months, and the median age at diagnosis is 4.7 months. Most patients begin to show symptoms within the first three months after birth, including limb floppiness, delayed motor development, feeding and swallowing difficulties, and labored breathing. Common physical examination findings in these patients include reduced muscle strength and tone, cardiomegaly, hepatomegaly, and macroglossia (5, 6). Genetic analysis of our patient revealed a homozygous mutation c.2662G>T (p.E888) in the GAA gene, a rare and pathogenic variant in IOPD. The significance of this study lies in its contribution to a deeper understanding of IOPD and a significant expansion of our knowledge of the clinical and molecular spectrum of PD.

### Methods for early screening of IOPD

Newborn screening (NBS) is the optimal method for early diagnosis and treatment of IOPD. NBS for Pompe disease includes the measurement of alpha-glucosidase (AαGLU) enzyme activity in dried blood spots (DBS) using fluorometric, tandem mass spectrometry, or digital microfluidic fluorometry techniques (7).

Traditionally, the diagnosis of IOPD involved analyzing AαGLU activity in lymphocytes, fibroblasts, and skeletal muscles. However, due to the presence of maltase-glucoamylase in neutrophils, measuring AαGLU activity in blood can lead to false-negative results. The use of acarbose, which inhibits maltase-glucoamylase activity, has enabled the implementation of large-scale NBS using DBS (8).

### Clinical characteristics and genetic analysis of IOPD

PD, or Glycogen Storage Disease Type II, is a hereditary metabolic disorder that can be divided into two main types based on the age of onset, the organs involved, and the speed of disease progression: Infantile-Onset Pompe Disease (IOPD) and Late-Onset Pompe Disease (LOPD) (9). IOPD usually presents within the first year of life and is characterized by a severe deficiency of the GAA. This deficiency leads to the accumulation of glycogen in skeletal and cardiac muscles, causing the symptoms of the disease. Serum muscle enzyme levels are a relatively simple and effective reference for the diagnosis of PD. In PD patients, about 95% of cases show elevated serum muscle enzyme creatine kinase levels, sometimes even up to 15 times the upper normal limit (10). Cardiac hypertrophy is a common initial presentation in PD patients, especially in those with IOPD. Studies show that IOPD patients with cardiac involvement typically present earlier and with more severe clinical symptoms. Therefore, regular echocardiographic examinations are needed in clinical practice to monitor changes in hypertrophic myocardium (11). In the cardiac imaging of PD patients, global myocardial hypertrophy can be observed, which progressively worsens over time. Initially, there may be left ventricular outflow tract obstruction, with or without accompanying normal left ventricular ejection fraction. However, in the later stages of the disease, left ventricular dilation may occur, accompanied by a significant decline in left ventricular ejection fraction (12). Monitoring these cardiac changes is crucial for the diagnosis and treatment progress of PD.

It has been reported that as many as 910 different mutations have been identified in the GAA gene. Of these, about 620 mutations, accounting for 68.1%, have been recognized as pathogenic to varying degrees, covering a spectrum from severe to potentially mild pathogenicity. Additionally, 290 mutations are categorized as likely benign, benign, or of uncertain significance. Among these, the c.1935C>A (p.D645E) mutation in the GAA gene is particularly common in mainland Chinese patients with IOPD. In patients with LOPD, the c.2238G>C (p.W746C) mutation is more prevalent (13). In our study, we observed a homozygous mutation c.2662G>T (p.E888) in the GAA gene in a child, with both parents being carriers of this mutation. This specific mutation leads to a change in the corresponding codon from a normal sequence to a stop codon, significantly altering the function of the protein.

**Figure 5:**
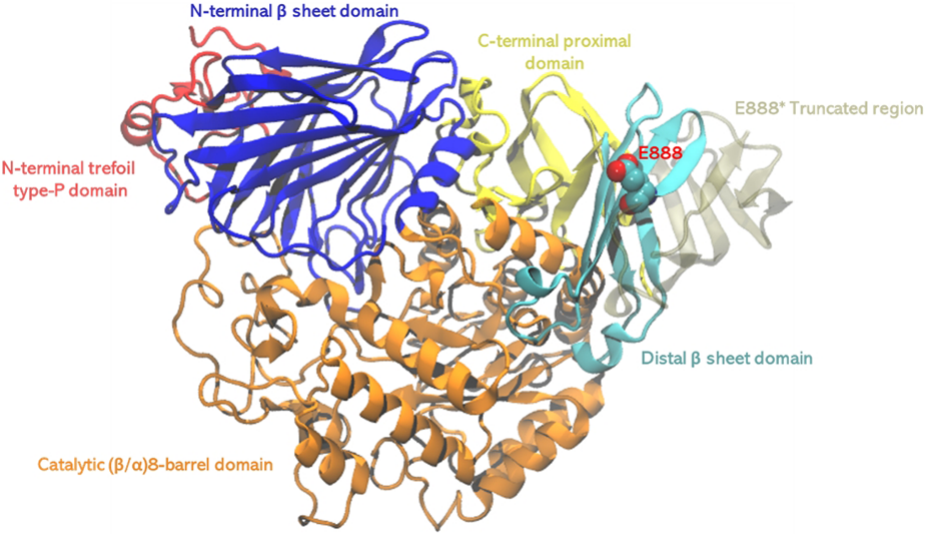
The structure of GAA, different domains were shown in different colors. The pathogenic stop codon mutation site E888 shown in spheres, and region 889–955 shown in transparent tan, which will be truncated with the stop codon mutation.

The overall structure of GAA comprises several domains: The N-terminal trefoil Type-P domain, a β-sheet domain, a catalytic (β/α)8-barrel domain with two inserts, and C-terminal proximal and distal β-sheet domains (14). The C-terminus, particularly the distal β-sheet domain, is thought to be crucial for the enzyme's structural integrity and proper localization to the lysosome (15). The E888 mutation will truncate a large portion of the C-terminal beta sheet, thus very likely destabilize the enzyme and affect the catalytic activities through allosteric effect (Figure 5). However, past studies, combined with in-silico simulations, have indicated the C-terminus domain may also serve as the binding site of the allosteric chaperone N-acetylcysteine, and the putative binding site located before the E888 site (16). This finding is not only important for understanding the genetic background of PD but also provides a basis for diagnosis.

### Potential link between PD and mitochondrial dysfunction

A recent study has revealed a potential link between PD and mitochondrial dysfunction (17). In this study, researchers differentiated induced pluripotent stem cells from PD patients into cardiomyocytes (PD-iCMs). These PD-iCMs exhibited typical PD cardiomyopathy characteristics, such as reduced GAA activity, abnormal glycogen accumulation in lysosomes, and hypertrophy of cardiomyocytes. Notably, these cardiomyocytes showed significant mitochondrial dysfunction, including a substantial reduction in the number of mitochondria, impaired respiratory function, and decreased ATP production. Part of these issues was attributed to an increase in intracellular ROS levels due to mitochondrial depolarization. Further analysis indicated that impaired mitochondrial fusion and autophagy, as well as a decrease in mitochondrial respiratory chain complex expression, were the primary causes of these mitochondrial dysfunctions. The study also found that supplementing with recombinant human GAA effectively improved mitochondrial function in these cardiomyocytes and alleviated heart pathology associated with PD. This discovery provides a new perspective for the treatment of PD, especially in how to alleviate disease symptoms by improving mitochondrial function. This research not only enhances our understanding of the relationship between PD and mitochondria but also provides an important biological basis for future treatment strategies.

Jeong and colleagues, in their study of PD in mouse and human models, have revealed significant findings regarding mitochondrial dysfunction (2). These findings include severe dysregulation of Ca^2+^ homeostasis, elevated levels of Ca^2+^ within mitochondria, a marked increase in ROS, a significant decrease in mitochondrial membrane potential, increased apoptosis via pathways independent of caspases, as well as a reduction in mitochondrial oxygen consumption and ATP production. These changes indicate that mitochondrial function is severely compromised in Pompe disease. Furthermore, through the analysis of gene expression in muscle cells of patients with PD, the research team discovered a significant upregulation of the β1 subunit of the L-type Ca^2+^ channel in these cells. This finding suggests that abnormalities in the function of Ca^2+^ channels may be a key element in the pathogenesis of Pompe disease. This study not only provides important evidence of Ca^2+^ homeostasis dysregulation and mitochondrial dysfunction in PD but also reveals that these changes could be early manifestations of a complex pathological process initiated by a single lysosomal enzyme deficiency, leading to severe and hard-to-treat autophagic myopathy. Most notably, the study found that L-type Ca^2+^ channel blockers, commonly used to treat other diseases such as heart conditions and hypertension, can reverse these mitochondrial dysfunctions in PD.

### Enzyme Replacement Therapy (ERT): current treatment strategy for PD

Enzyme Replacement Therapy (ERT) is currently the only proven effective specific treatment for PD and can be used for patients of all ages, including those with IOPD and LOPD. While patients with PD receive ERT, the involvement of multidisciplinary teams is crucial, including respiratory system management, physical rehabilitation therapy, gastrointestinal and nutritional management, orthopedic and spinal surgical management, circulatory system management, and other supportive treatments (18, 19).

A major limitation in our case was that despite early diagnosis, the family refused ERT due to economic constraints. Therefore, it was not possible to further observe the child's response to ERT and the outcome, and follow-up information about the patient's condition was not successfully obtained.

Given the significant variability in the clinical presentation of IOPD, this study highlights the importance of measuring GAA enzyme activity and analyzing the GAA gene for accurate diagnosis, aiding clinicians in early identification and diagnosis of IOPD. The discovery of uncommon myocardial disease in IOPD in this study suggests that clinicians should regularly conduct echocardiographic examinations in IOPD patients to monitor changes like cardiac hypertrophy. The potential connection between PD and mitochondrial dysfunction may prompt clinicians to consider improving mitochondrial function in the treatment of PD.

Due to the high cost of ERT, future research should focus on developing more affordable treatment strategies to improve the quality of life for patients. The association between mitochondrial dysfunction and PD discovered in this study offers a new perspective. Future research could explore how to alleviate PD symptoms by improving mitochondrial function. As there are numerous mutations in the GAA gene, a deeper investigation into how different mutations affect the disease phenotype and their impact on treatment response will be an important direction for future research.

However, limitations of our study include its reliance on a single case, which may not provide a comprehensive view of the disease's variability. This small sample size limits the generalizability of the findings to the broader population of patients with IOPD. Additionally, the article mentions the lack of follow-up information on the patient's condition after discharge, which restricts the ability to observe the long-term effects of the disease and the treatments used.

## Conclusion

The clinical presentation of IOPD shows significant variability, which makes its diagnosis challenging. In diagnosing this condition, measuring GAA enzyme activity and analyzing the GAA gene are crucial diagnostic tools. These tests help confirm the presence of the disease and guide subsequent treatment. Although Enzyme Replacement Therapy has been proven to significantly improve the quality of life for patients, its widespread adoption is hindered by its high cost. The expensive price of the medication is a major barrier to the promotion of this treatment, especially in resource-limited regions. Therefore, finding more economical treatment options or reducing the cost of existing treatments is essential to improve the quality of life for patients.

## Data Availability

The original contributions presented in the study are included in the article/supplementary materials, further inquiries can be directed to the corresponding author.

## References

[1] Bellotti AS Andreoli L Ronchi D Bresolin N Comi GP Corti S. Molecular approaches for the treatment of Pompe disease. Mol Neurobiol. (2020) 57:1259–80. 10.1007/s12035-019-01820-5 31713816

[2] Lim JA Li L Kakhlon O Myerowitz R Raben N. Defects in calcium homeostasis and mitochondria can be reversed in Pompe disease. Autophagy. (2015) 11:385–402. 10.1080/15548627.2015.1009779 25758767 PMC4502791

[3] Lopaschuk GD Karwi QG Tian R Wende AR Abel ED. Cardiac energy metabolism in heart failure. Circ Res. (2021) 128:1487–513. 10.1161/CIRCRESAHA.121.318241 33983836 PMC8136750

[4] Lucas A de Lacerda AJ Araújo M David C Ponte VY Coelho BN Diazoxide modulates cardiac hypertrophy by targeting h2o2 generation and mitochondrial superoxide dismutase activity. Curr Mol Pharmacol. (2020) 13:76–83. 10.2174/1874467212666190723144006 31340743

[5] Martínez M Romero MG Guereta LG Cabrera M Regojo RM Albajara L Infantile-onset Pompe disease with neonatal debut. Medicine (Baltimore). (2017) 96:e9186. 10.1097/MD.0000000000009186 29390460 PMC5758162

[6] Chan M Jalil JA Yakob Y Wahab SAA Ali EZ Khalid MKNM Genotype, phenotype and treatment outcomes of 17 Malaysian patients with infantile-onset Pompe disease and the identification of 3 novel gaa variants. Orphanet J Rare Dis. (2023) 18:231. 10.1186/s13023-023-02848-6 37542277 PMC10403872

[7] Karadağ GA Gürel S. To detect potential pathways and target genes in infantile Pompe patients using computational analysis. Bioimpacts. (2022) 12:89–105. 10.34172/bi.2022.23467 35411297 PMC8905584

[8] Sawada T Kido J Nakamura K. Newborn screening for Pompe disease. Int J Neonatal Screen. (2020) 6:31. 10.3390/ijns6020031 33073027 PMC7423004

[9] Zhang H Chen J Zhu Y Ma X Zhong W. Case report: identification of compound heterozygous mutations in a patient with late-onset glycogen storage disease type ii (Pompe disease). Front Neurol. (2022) 13. 10.3389/fneur.2022.839263 PMC8977516 35386406

[10] Hahn A Hennermann JB Huemer M Kampmann C Marquardt T Mengel E Diagnosis and care of infants and children with Pompe disease. Klin Padiatr. (2020. 10.1055/a-1110-7335 32069498

[11] van Kooten HA Roelen C Brusse E van der Beek N Michels M van der Ploeg AT Cardiovascular disease in non-classic Pompe disease: a systematic review. Neuromuscul Disord. (2021) 31:79–90. 10.1016/j.nmd.2020.10.009 33386209

[12] Boentert M Florian A Dräger B Young P Yilmaz A. Pattern and prognostic value of cardiac involvement in patients with late-onset Pompe disease: a comprehensive cardiovascular magnetic resonance approach. J Cardiovasc Magn Reson. (2016) 18:91. 10.1186/s12968-016-0311-9 27931223 PMC5146906

[13] Liu X Wang Z Jin W Lv H Zhang W Que C Clinical and gaa gene mutation analysis in mainland Chinese patients with late-onset Pompe disease: identifying c.2238g>c as the most common mutation. BMC Med Genet. (2014) 15. 10.1186/s12881-014-0141-2 PMC4411720 25526786

[14] Roig-Zamboni V Cobucci-Ponzano B Iacono R Ferrara MC Germany S Bourne Y Structure of human lysosomal acid α-glucosidase-a guide for the treatment of Pompe disease. Nat Commun. (2017) 8:1111. 10.1038/s41467-017-01263-3 29061980 PMC5653652

[15] Moreland RJ Jin X Zhang XK Decker RW Albee KL Lee KL Lysosomal acid alpha-glucosidase consists of four different peptides processed from a single chain precursor. J Biol Chem. (2005) 280:6780–91. 10.1074/jbc.M404008200 15520017

[16] Porto C Ferrara MC Meli M Acampora E Avolio V Rosa M Pharmacological enhancement of α-glucosidase by the allosteric chaperone n-acetylcysteine. Mol Ther. (2012) 20:2201–11. 10.1038/mt.2012.152 22990675 PMC3519985

[17] Huang W Zhou R Jiang C Wang J Zhou Y Xu X Mitochondrial dysfunction is associated with hypertrophic cardiomyopathy in Pompe disease—specific induced pluripotent stem cell-derived cardiomyocytes. Cell Prolif. (2023):e13573. 10.1111/cpr.13573 37916452 PMC10984102

[18] Llerena JJ Nascimento OJ Oliveira AS Dourado JM Marrone CD Siqueira HH Guidelines for the diagnosis, treatment and clinical monitoring of patients with juvenile and adult Pompe disease. Arq Neuropsiquiatr. (2016) 74:166–76. 10.1590/0004-282X20150194 26690841

[19] Tarnopolsky M Katzberg H Petrof BJ Sirrs S Sarnat HB Myers K Pompe disease: diagnosis and management. Evidence-based guidelines from a Canadian expert panel. Can J Neurol Sci. (2016) 43:472–85. 10.1017/cjn.2016.37 27055517

